# Improved Annotations of 23 Differentially Expressed Hypothetical Proteins in Methicillin Resistant S.aureus

**DOI:** 10.6026/97320630013104

**Published:** 2017-04-30

**Authors:** Jessica Marklevitz, Laura K. Harris

**Affiliations:** 1 Department of Science, Davenport University, Lansing, Michigan, United States of America;; 2 Department of Health Informatics, Rutgers School of Health Professions, Newark, New Jersey, United States of America;

**Keywords:** Annotations, Hypothetical proteins, Methicillin, S.aureus

## Abstract

Antibiotic resistant Staphylococcus aureus is a major public health concern effecting millions of people annually. Medical science has documented completely untreatable S. aureus infections. These strains are appearing in the community with increasing frequency. New diagnostic and therapeutic options are needed to combat this deadly infection. Interestingly, around 50% of the proteins in S. aureus are annotated as hypothetical. Methods to select hypothetical proteins related to antibiotic resistance have been inadequate. This study uses differential gene expression to identify hypothetical proteins related to antibiotic resistant phenotype strain variations. We apply computational tools to predict physiochemical properties, cellular location, sequence-based homologs, domains, 3D modeling, active site features, and binding partners. Nine of 23 hypothetical proteins were <100 residues, unlikely to be functional proteins based on size. Of the 14 differentially expressed hypothetical proteins examined, confident predictions on function could not be made. Most identified domains had unknown functions. Six hypothetical protein models had >50% confidence over >20% residues. These findings indicate the method of hypothetical protein identification is sufficient; however, current scientific knowledge is inadequate to properly annotate these proteins. This process should be repeated regularly until entire genomes are clearly and accurately annotated.

## Background

Antibiotic therapy has been the marvel of modern medicine since the advent of Penicillin in the 1920s. Over seventy billion doses of antibiotics are consumed globally each year [1]. Antibiotics are a low-cost resource to treat food-borne and other sanitation-related infections that commonly affect poor people. Among wealthier countries, antibiotics play a pivotal role as a prophylactic, controlling infections associated with medical practices such as surgery [1,2]. Unfortunately, this usage exposes normal microbial flora to anti-bacterial drugs, allowing them to develop resistances so the drugs lose effectiveness. Medical science has been unable to cultivate new antibiotics as fast as resistances to current therapies are rising [2,3]. Infectious organisms that are resistant to every antibiotic developed have been reported. This antibiotic resistance crisis is a critical challenge for humanity’s medical future.

Staphylococcus aureus, an opportunistic pathogen that was originally associated with hospital-acquired infections, was the first organism to show resistance to Penicillin and its synthetic offspring like Methicillin. Though hospital-acquired Methicillinresistant S. aureus (MRSA) cases proliferated through the late 20th century, recent years have seen decreases in the number of hospital-acquired MRSA infections due to improvements in sanitation procedures and increases in Vancomycin use despite its potential side effects [4]. Unfortunately, community-acquired MRSA infections have dominated recently since over 100 million people harbor MRSA strains as part of normal skin flora according to Dutch and United States prevalence data [5]. Therefore, the United States Center for Disease Control lists MRSA and Vancomycin-resistant S. aureus (VRSA) strains as serious and concerning public health threats, respectively, estimating over 80,000 invasive MRSA infections with 20,000 related deaths annually, many in immuno-compromised patients including children [5]. While there are no S. aureus strains currently resistant to all antibiotics, completely resistant strains of other infectious organisms have emerged so the same outcome will likely befall S. aureus soon.

A challenge to developing new antibiotic therapies is genome annotation. Around 50% of proteins identified in the S. aureus genome are annotated as hypothetical [6,7]. At annotation, hypothetical proteins are predicted by sequence only and lack homology to known proteins. Researchers further define hypothetical proteins by their larger than 100 amino acids size, since smaller sequences likely represent other macromolecular structures such as short interfering RNA (siRNA) rather than functional proteins [8]. True hypothetical proteins have similar features to other hypothetical proteins due to lack of experimental evidence to predict function for the protein family, though frequently hypothetical proteins found in databases represent old genome annotations in need of update. Several studies have used various methods to identify hypothetical proteins related to antibiotic resistance in S. aureus. Early studies randomly selected hypothetical proteins for characterization [6,7, 9,10]. While this approach developed and demonstrated computational procedures that contribute to hypothetical protein characterization, it is limited in its ability to identify hypothetical proteins specifically connected to antibiotic resistance. To improve the selection process, we formerly developed crossspecies approach that used proteins with experimentally established structures from the major facilitator superfamily; a large, highly conserved protein family associated with antibiotic resistance [7]. This approach worked because of the large percentage of hypothetical proteins in the S. aureus genome, but it becomes inadequate if a hypothetical protein related to resistance has no well-characterized homolog in another species, a common challenge for hypothetical proteins. Better methods for identifying antibiotic resistant-related hypothetical proteins are needed.

Microarray and other forms of publicly accessible gene expression data can provide an excellent repository for targeted identification of resistance linked hypothetical proteins in S. aureus. For example, Ham and colleagues examined mRNA expression between antibiotic resistant (MRSA; ATCC 33591, shown to be susceptible only to Vancomycin and Kanamycin) and sensitive (MSSA; ATCC 25923) strains using Affymetrix GeneChip® technology [11]. They statistically compared mRNA expression levels between the strains to uncover potential mechanisms of resistance, but did not consider hypothetical proteins. This presents an opportunity to characterize hypothetical proteins whose differential expression constitutes a drug-resistant genomic background.

This study uses computational procedures to characterize statistically significant differentially expressed hypothetical proteins from the microarray data generated by Ham and associates. By comparing natural gene expression between antibiotic sensitive and resistant strains, new insight into strain background differences is gained. These variations could uncover new resistance mechanisms, further developing into a useful diagnostic tool or potential antibiotic therapeutic target. This would improve outcomes for patients infected with MRSA strains through faster and more effective treatment options.

## Methodology

Normalized mRNA expression data from Ham’s study is available at the National Center for Biotechnology Information’s (NCBI) Gene Expression Omnibus (GEO; Dataset Record GDS4242; GEO accession GSE18289) [11]. Data consisted of 7774 entries, each with probe name and six samples representing triplicates of both MSSA (ATCC 25923) and MRSA (ATCC 33591) strains. Probe names were converted to gene names and descriptions per Affymetrix chip platform and non-hypothetical proteins were removed. Excel calculated T-scores and p-values based on Student’s T-test two-tailed, equal variance formulas. The study rejected hypothetical proteins with a p>0.05 as these were not differentially expressed. The National Center for Biotechnology Information (NCBI) and UniProt databases confirmed hypothetical protein annotation.

This study used numerous algorithms to characterize these hypothetical proteins and default program settings were used for all analyses. ExPASy’s Protparam server calculated physiochemical properties including number of amino acids, molecular weight, positively and negatively charged residues, theoretically isoelectric point (pI), extinction coefficient, aliphatic index (AI), instability index (II), and the grand average hydropathy (GRAVY) [12]. By hypothetical protein definition, those identified through differential expression yet smaller than 100 amino acids were excluded from further study.

PSortB and SOSUI servers predicted each hypothetical protein’s cellular location. PSortB predicted between cytoplasm, cytoplasmic membrane, cell wall, or extracellular locations [13]. SOSUI calculated transmembrane regions and solubility indices, a valuable confirmation of PSortB predictions [14]. These complementary algorithms provide confidence for cellular localization estimates.

Sequence similarity and domain identification projected functional features of hypothetical proteins. The Position-Specific Iterative (PSI) Basic Local Alignment Search Tool (BLAST) identified potential homologs from the NCBI database based on protein sequence similarities. Further, both Conserved Domain Database (CDD) BLAST and Pfam algorithms predicted potential domains within each hypothetical protein. CDD-BLAST uses a PSI-BLAST variation to identify domains by comparison of the protein sequence’s position specific scoring matrix to those in the NCBI database [15]. Alternatively, Pfam is a separately curated database of Hidden Markov Models and multiple sequence alignments representing protein domain families [16]. These complementary approaches provide a level of validation to this study’s findings.

For model development and characterization, we used the integrated Phyre2 and 3DLigandSite servers. Phyre2 produced a tertiary structure model, predicted ligand-binding sites, and analyzed the effect of amino acid variants through automatic homology detection methods [17]. Phyre2’s model advanced to 3DLigandSite for active site characterization and docking predictions. 3DLigandSite identifies homologous structures with bound ligands by searching a structural library then superimposing those ligands onto the Phyre2’s protein structure [18]. Together, Phyre2 and 3DLigandSite servers modeled the protein and characterized its binding site.

The Search Tool for Interactions of Chemicals (STITCH) database predicted potential ligand interactions for each hypothetical protein. STITCH draws upon scientific literature and several databases, including the formerly separate Search Tool for the Retrieval of Interacting Genes/Proteins (STRING) database, which houses high-throughput experiment and conserved coexpression data, to calculate drug-target interactions, binding affinities, and biological pathways [19]. STITCH is a useful tool to predict protein and chemical binding partners.

## Results

The mRNA expression dataset, GSE18289, was downloaded from GEO and Excel calculated the T-statistic and p-value for each protein. Twenty-seven proteins labeled as hypothetical in NCBI, 16 and 11 up- and down regulated in MRSA, respectively, had <0.05 p-values. Four of these proteins had predicted functions in UniProt, an endotoxin (SACOL0468, up regulated, T-score 9.00), exotoxin (SACOL1178, up regulated, T-score 10.17), phosphate dikinase regulatory protein (SACOL1620, down regulated, Tscore -9.80), and a lipoprotein (SACOL1531, up regulated, T-score 7.89). Since these proteins had predicted identities, they were excluded from further study. The remaining 23 proteins are listed by T-score in Table 1.

Few hypothetical proteins had well defined homologs in the NCBI database as identified by PSI-BLAST (Table 4). Most top homologs came from S. aureus and were vaguely annotated or had low sequence similarity to the hypothetical protein. Three proteins, SACOL0323, SACOL2481, and SACOL0710, had homologs from other species, Mucilaginibacter, Helicobacter mustelae, and Bacillus cereus, respectively. Interestingly, four hypothetical proteins had membrane protein for their top homolog. PSortB and SOSUI confirm that SACOL0109, SACOL0075, and SACOL2241 are likely membrane proteins too (Table 2 and 3, respectively). However, according to these algorithms, SACOL2481 is a soluble, cytoplasmic protein. Further, PSortB predicted SACOL0488 to reside in the cytoplasm, which PSI-BLAST’s top homolog confirmed, though PSortB was unable to confirm extracellular locations for SACOL0835 and SACOL0267 where top homologs are exported proteins. Interestingly, for SACOL2123, PSI-BLAST identified its top homolog as a PF11042 family member. This matched CDDBLAST domain identification of pfam11042, showing the interconnectivity of these computational tools, and was confirmed by Pfam itself.

**Figure 2:**
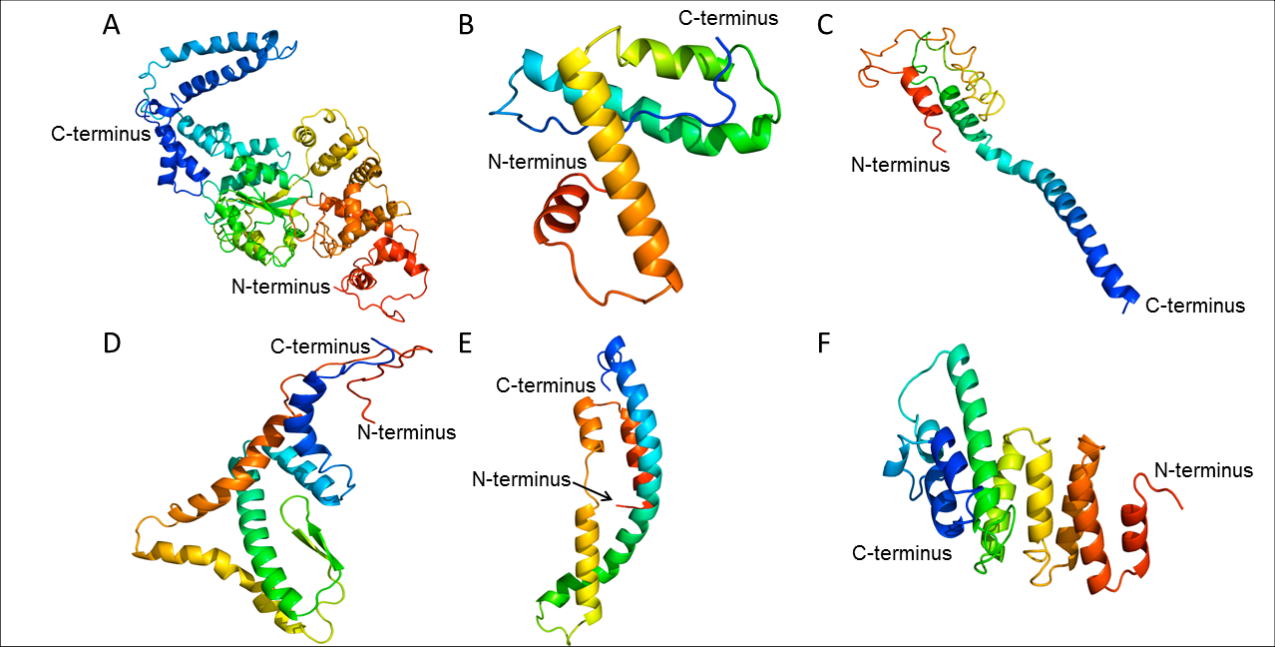
Phyre2’s intensive mode models for hypothetical proteins SACOL1859 (A), SACOL0323 (B), SACOL0109 (C), SACOL1956 (D), SACOL0488 (E), and SACOL0710 (F). Image colored by rainbow N- to C-terminus.

Phyre2 and 3DLigand servers performed hypothetical protein modeling and active site characterization. Similarity measurements of the hypothetical protein target to its experimental structure template are in Table 7. These findings represent Phyre2 running in normal mode. Hypothetical proteins with coverage >25% in normal mode were re-run under Phyre2’s intensive mode with the results show in Figure 2. Remarkably, under this mode, SACOL1859 and SACOL0710 models had 88% and 89% residues modeled with >90% confidence. No amino acids from the other four proteins could be modelled with that confidence. Unfortunately, 3DLigand was unable to make a prediction for any hypothetical protein examined in this study due to insufficient homologous structures with ligands bound.

STITCH predicted binding partners for hypothetical proteins. STITCH was unable to predict binding partners for the following hypothetical proteins: SACOL2481, SACOL0835, and SACOL2241. Most top binding partners were fellow hypothetical proteins with confidence scores listed in Table 8. This implies that more database annotation and/or wet bench work are needed to fully understand how these proteins work. SACOL0323, SACOL2123, and SACOL0710 had top matching binding partners that were not hypothetical proteins.

SACOL0323 matched a prophage L54a, Cro-like protein. SACOL2123 had equal scores to a M20/M25/M40 family peptidase (SACOL2125) and a hypothetical protein (SACOL2124). SACOL0710 equally matched a phosphotransferase mannose-specific family component IIA (SACOL0709) and a DAK2 domain-containing protein (SACOL0708). These results did not correlate with the findings from other programs used in this study.

## Conclusion

Antibiotic resistance is a major global health crisis. Infections, like those caused by Methicillin-resistant S. aureus, are becoming untreatable, and increasing fatalities from these once curable diseases. Faster techniques to identify drug-resistant organisms and new therapeutics are needed to improve patient outcomes. Characterizing hypothetical proteins, particularly those contributing to resistance, may hold the key to unlock this health predicament. This work provides insight into hypothetical proteins related to antibiotic resistance, potentially leading to improved diagnostic tools and therapeutics against antibiotic resistant S. aureus. It characterized differentially expressed hypothetical proteins between Methicillin-sensitive and resistant strains whereas other studies have randomly selected or performed cross-species comparisons to identify hypothetical proteins of interest. Our approach to identify hypothetical proteins related to antibiotic resistance is an improvement over prior methods. However, computational algorithms were unable to confidently predict functions for any of the 14 differentially expressed hypothetical proteins examined. Most programs struggled to identify parameters, such as domains or binding partners. Those that were found usually had unknown functions or little sequence homolog. These results indicate that using statistically significant differential expression from a publically available antibiotic resistant strain comparison microarray study will identify proteins potentially related to antibiotic resistance for which more scientific knowledge is needed.

## Figures and Tables

**Table 1:** Differential expression T-scores and physiochemical properties of 23 hypothetical proteins

Protein	T-score	# AA	MW	pI	# neg	# pos	EC	II	AI	GRAVY
SACOL0919	18.77	45	5270	9.03	4	6	2980	17.29	136.22	0.56
SACOL1859	12.23	1016	120681	5.7	147	130	161360	37.5	95.75	-0.415
SACOL1346	10.83	64	7573	4.1	16	6	5960	21.26	92.81	-0.42
SACOL0356	9.29	78	8726	4.32	17	7	5960	41.83	86.28	-0.529
SACOL0326	7.48	74	8841	4.54	18	7	11460	52.27	77.7	-0.938
SACOL0323	7.32	102	11944	7.91	17	18	9970	33.41	89.8	-0.762
SACOL0109	6.83	135	15123	4.45	14	8	26930	39.15	132.89	0.757
SACOL0087	6.62	35	4172	6	5	5	4470	25.62	94.57	-0.149
SACOL0075	6.04	200	22662	9.55	9	20	31860	42.51	121.35	0.665
SACOL0644	5.35	208	24690	9.55	14	25	43890	29.04	125.48	0.448
SACOL0350	3.8	118	13923	10.08	14	28	12950	26.44	76.02	-0.804
SACOL0362	3.77	66	7806	8.03	8	9	9970	37.99	125.45	0.185
SACOL2481	3.23	121	14067	4.54	21	11	5960	35.52	118.43	-0.098
SACOL0835	-2.56	209	24070	9.07	31	36	8940	64.82	34.16	-1.974
SACOL2241	-6.45	129	14638	9.73	3	8	18450	26.53	155.74	1.209
SACOL2123	-6.59	223	25856	4.74	43	28	28550	41.8	93	-0.289
SACOL2491	-8.97	63	7221	4.6	10	6	7450	25.52	97.46	-0.146
SACOL2571	-9.8	63	7266	5.44	9	7	1490	17.06	103.65	-0.233
SACOL2076	-10.78	45	5070	10.46	3	10	1	45.35	114.67	-0.424
SACOL1956	-14.64	176	20513	9.25	10	15	21555	39.83	132.95	0.747
SACOL0267	-15.31	507	57978	8.02	93	95	36790	23.76	74.48	-0.906
SACOL0488	-24.11	107	13458	5.23	26	21	15930	65.54	59.25	-1.693
SACOL0710	-25.02	165	19009	5.08	27	17	10430	33.94	100.48	-0.181
# AA, number of amino acids; MW, molecular weight; pI, theoretical isoelectric point; # neg, total number of negatively charged residues (Asp + Glu); # pos, total number of positively charged residues (Arg + Lys); EC, extinction coefficient assuming all pairs of Cys residues form cystines; II, instability index; AI, aliphatic index; GRAVY, grand average hydropathy. 1As there are no Trp, Tyr, or Cys in the region considered, protein should not be visible by UV spectrophotometry.									

**Table 2:** PSortB cellular location of 14 hypothetical proteins

Protein	Location	Localization Score
SACOL1859	Unknown	2.501
SACOL0323	Cytoplasm	7.5
SACOL0109	Cytoplasmic membrane	10
SACOL0075	Cytoplasmic membrane	10
SACOL0644	Cytoplasmic membrane	10
SACOL0350	Unknown	2.501
SACOL2481	Cytoplasm	7.5
SACOL0835	Cytoplasmic membrane	9.55
SACOL2241	Cytoplasmic membrane	10
SACOL2123	Cytoplasm	7.5
SACOL1956	Cytoplasmic membrane	10
SACOL0267	Unknown	3.332
SACOL0488	Cytoplasm	7.5
SACOL0710	Cytoplasm	7.5
^1^Equal probability of the protein being located in any cellular structure: cytoplasm, cytoplasmic membrane, cell wall, or extracellular. 2Equal probability of protein being located in cytoplasmic membrane, cell wall, or extracellular.		

**Table 3:** SOSUI results for 7 transmembrane hypothetical proteins

Protein	N-terminal	Transmembrane Region	C-terminal	Type	Length
SACOL0109	53	IGKIAIWIGIVAQIYFSVVFVRM	75	PRIMARY	23
	89	IFLLGLILALFTVLPTIFTAIYM	111	PRIMARY	23
	123	IVYAIIALCLYNFLSSILWLIGG	145	PRIMARY	23
SACOL0075	7	KIAIWIGIVAQIYFSVVFVRMIS	29	PRIMARY	23
	41	IFLLGLILALFTVLPTIFTAIYM	63	PRIMARY	23
	75	IVYAIIALCLYNFLSSILWLIGG	97	PRIMARY	23
SACOL0644	23	YLLIDLVSTWLVYFFPFINWFIP	45	SECONDARY	23
	94	QLDNKILISLCFIGFIGIAAFYI	116	PRIMARY	23
	147	SFIVFTYLLLGGCSILFLIWLMT	169	PRIMARY	23
	174	NLLVFIMWIIITIFFFLISMGSI	196	PRIMARY	23
SACOL0835	23	AKVVSIATVLLLLGGLVFAIFAY	45	PRIMARY	23
SACOL2241	10	ALIGIFLILCEFFYGIPFLGATF	32	PRIMARY	23
	40	PLLFNALLYLILTIILLVNRQNA	62	PRIMARY	23
	65	PIAIIPIFGIVGSFLAIIPFLGI	87	PRIMARY	23
	90	HWILFFLMILFVLVVLSAPTYIP	112	PRIMARY	23
SACOL1956	16	FIILQLVIALFVILFTYKWALGV	38	PRIMARY	23
	50	LVYGFAGFIILLILHELIHRALF	72	PRIMARY	23
	103	QFSIIMLSPLILLSTGLLILIKV	125	PRIMARY	23
	134	MFSMHTAYCFIDILLVALTISSS	156	PRIMARY	23
SACOL0267	6	KIIIPIIIVLLLIGGIAWGVYAF	28	PRIMARY	23

**Table 4:** Top PSI-BLAST result for 14 hypothetical proteins

Protein	PSI-BLAST Match	Query Cover	E-value	Identity
SACOL1859	NTPase	100%	0	100%
SACOL0323	Metallophosphoesterase	59%	1.6	31%
SACOL0109	Membrane protein	100%	3.00E-44	59%
SACOL0075	Membrane spanning protein	90%	7.00E-124	98%
SACOL0644	tandem five-TM protein	100%	1.00E-143	99%
SACOL0350	Phage protein	100%	5.00E-80	98%
SACOL2481	Outer membrane protein	59%	4.3	27%
SACOL0835	Exported protein	91%	8.00E-128	100%
SACOL2241	Membrane protein	79%	3.00E-64	100%
SACOL2123	PF11042 family protein	100%	1.00E-91	65%
SACOL1956	Permease	100%	3.00E-123	100%
SACOL0267	Exported protein	51%	8.00E-170	98%
SACOL0488	Cytosolic protein	89%	2.00E-59	100%
SACOL0710	RHS repeat-associated core domain-containing protein	87%	1.00E-09	29%

**Table 5:** CDD-BLAST domain data for 7 hypothetical proteins

Protein	Domains	Description	E-value
SACOL1859	pfam13401	AAA	1.61E-04
	smart00382	ATPase	7.59E-03
SACOL0644	pfam04276	Protein of unknown function (DUF443)	1.03E-37
SACOL0350	pfam07768	PVL ORF-50-like family	8.79E-47
SACOL0835	pfam16228	Domain of unknown function (DUF4887)	1.18E-12
SACOL2123	pfam11042	Protein of unknown function (DUF2750)	2.38E-20
SACOL1956	pfam11667	Putative zincin peptidase	4.49E-08
SACOL0488	pfam13654	AAA	5.17E-03

**Table 6:** Pfam domain data for 5 hypothetical proteins

Protein	Domain	Description	E-value
SACOL0644	DUF443	Unknown function	9.80E-56
SACOL0350	PVL_ORF50	Panton-Valentine leucocidin ORF-50- like family	2.80E-45
SACOL0835	DUF4887	Unknown function	1.70E-50
SACOL2123	DUF2750	Unknown function	2.10E-21
SACOL1956	DUF3267	Putative zincin peptidase	1.60E-18

**Table 7:** Phyre2 model data for 14 hypothetical proteins

Protein	Template	Template Description	Confidence	Coverage
SACOL1859	c4kxfF	nlr family card domain-containing protein 4	99.70%	30%
SACOL0323	d1nu9c1	immunoglobulin/albumin-binding domain-like	37.80%	25%
SACOL0109	c3x29A	crystal structure of mouse claudin-19	73.20%	45%
SACOL0075	c4zxsD	virion egress protein ul31	55.60%	20%
SACOL0644	c4yjxB	ATP-dependent clp protease adapter protein	30.70%	7%
SACOL0350	c2qdqA	talin-1	40.70%	21%
SACOL2481	c3daoB	putative phosphatse	23.30%	17%
SACOL0835	c2ifmA	pf1 filamentous bacteriophage	80.30%	14%
SACOL2241	c2ap8A	bombinin h4	43.20%	10%
SACOL2123	c1zctB	glycogenin-1	46.30%	12%
SACOL1956	c3b4rB	putative zinc metalloprotease mj0392	89.30%	41%
SACOL0267	c3jcuj	photosystem ii reaction center protein j	50.40%	5%
SACOL0488	c4c46B	general control protein gcn4	80.60%	29%
SACOL0710	c1kt0A	lare fkbp-like protein, fkbp51, involved in steroid2 receptorcomplexes	93.60%	47%

**Table 8:** Top STITCH predicted binding partners for 11 hypothetical proteins

Protein	Substrate	Score
SACOL1859	SACOL1860	0.651
SACOL0323	SACOL0322	0.819
SACOL0109	SACOL0110	0.692
SACOL0075	SACOL0076	0.462
SACOL0644	SACOL0643	0.859
SACOL0350	SACOL0351	0.859
SACOL2123	SACOL2125	0.422
	SACOL2124	0.422
SACOL1956	SACOL2519	0.685
SACOL0267	SACOL0266	0.694
SACOL0488	SACOL0487	0.859
	SACOL0486	0.859
SACOL0710	SACOL0709	0.57
	SACOL0708	0.57

**Figure 1:**
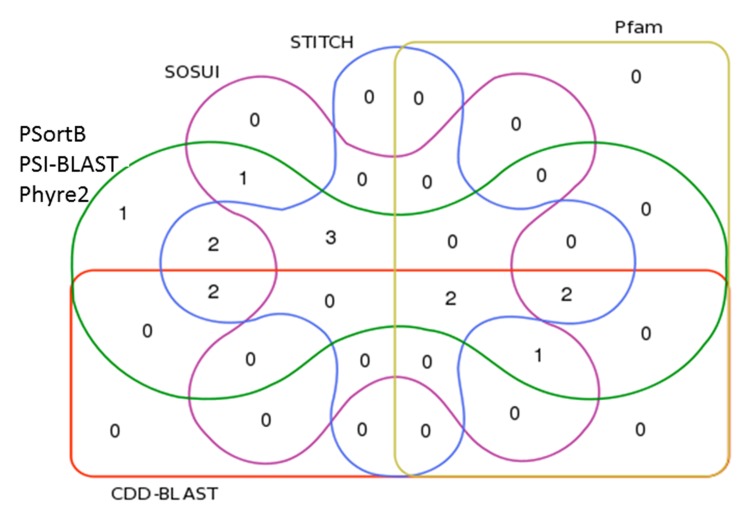
Venn diagram illustrating overlap of study evalations. PSortB, PSI-BLAST, and Phyre2 (green line) characterized all 14 hypothetical proteins that passed Expasy’s size exclusion criteria. Only those algorithms found results for SACOL2481 (1). SACOL2241 also had a SOSUI (purple line) result (1). SACOL0710 and SACOL0323 had STITCH (blue line) results (2). SACOL0488 had both STITCH and CDD-BLAST (orange line) results (2). SACOL0267, SACOL0109, and SACOL0075 had SOSUI and STITCH results (3). SACOL2123 and SACOL0350 had CDD-BLAST, Pfam (yellow-line), and STITCH results (2). SACOL1956 and SACOL0644 had results from all programs (2) and SACOL0835 had results from all except STITCH (1).
